# An investigation into the spatial patterns of invasive common milkweed (*Asclepias syriaca* L.) stands through the utilization of drone images

**DOI:** 10.1038/s41598-025-14034-8

**Published:** 2025-08-07

**Authors:** László Bakacsy, Tomás Zakar

**Affiliations:** 1 Department of Plant Biology, University of Szeged, Közép fasor 52, Szeged, 6726 Hungary; 2 Institute of Photonics and Electronics, Czech Academy of Sciences, Chaberská 1014/57 182 52 Praha 8, Kobylisy, Czech Republic

**Keywords:** Conservation strategies, Invasive plant, Sand dune vegetation, Spatial analysis, Spatial point pattern, Unmanned aerial vehicles (UAVs), Ecology, Conservation biology, Grassland ecology, Invasive species

## Abstract

The phenomenon of biological invasions represents one of the most significant threats to biodiversity. A fundamental aspect of combating invasive plant species is the comprehension of the spatial and temporal alterations in their population dynamics. One of the important habitats of the European Union is the Pannon sand grasslands in Hungary, which are primarily threatened by the invasive common milkweed (*Asclepias syriaca*). The objective of this study was to ascertain the efficacy of drone imaging in examining the spatial patterns of milkweed shoots in comparison to ground survey data. To facilitate comparison, a survey was conducted on 12 milkweed populations in the Fülöpháza area of Kiskunság National Park. In each population, a 12-meter transect (comprising six contiguous 2 m × 2 m quadrats) was designated within which the positions of the shoots were recorded with centimeter accuracy through ground surveys. The individual shoots were marked on images captured from an altitude of 20 m using a drone. The results indicated that the number of shoots identified in the drone images was slightly lower than in the ground surveys; however, a positive correlation was observed between the two datasets (*r* = 0.9594). A strong positive correlation was evident between the ground and drone surveys in terms of both the average distance between shoots and the observed pattern (*r* = 0.933 and *r* = 0.9146). In light of these findings, it can be concluded that drone imaging represents an effective method for examining the size and pattern of populations. Consequently, it may prove to be a valuable tool for the accurate planning of invasive species management in conservation efforts and the monitoring of the effectiveness of treatments.

## Introduction

Invasive species introduced from other geographical regions can disrupt local ecosystems. When they enter new habitats, they often outcompete native plants and animals, leading to serious environmental harm. Biological invasions represent one of the most significant contemporary threats to biodiversity^1–3^. The most critical element in the preventing the spread of invasive species is the rapid identification and prevention of their introduction. Eradication or isolation represents the most effective means of addressing established populations. Nevertheless, the success of these strategies hinges on the accurate monitoring of invasive species populations^4,5^.

Understanding the spatial organization of vegetation and plant species plays a critical role in ecological research. It provides essential insights into ecological dynamics, supports biodiversity assessment, and aids in monitoring ecosystem health^6^. Such spatial configurations reflect the intricate relationships between plants and their environment, shaped by processes such as competition, facilitation, and environmental gradients. At the local scale, spatial patterns provide insights into the mechanisms underlying species coexistence, including niche differentiation and habitat preferences^7^. Moreover, they impact the structure and composition of communities by influencing the distributions and abundances of species across landscapes. At broader scales, spatial patterns contribute to landscape-level processes such as connectivity and fragmentation. An understanding of these patterns facilitates the development of effective conservation strategies, as it enables the identification of key habitats, corridors, and areas of high ecological value. Furthermore, spatial patterns in vegetation serve as indicators of environmental changes, including those resulting from human activities such as land use change and climate change. Changes in vegetation can serve as indicators of shifts in ecosystem functions and services. Consequently, it is essential to employ remote sensing (RS) technologies to monitor spatial patterns, thereby facilitating the development of effective conservation and management strategies^6,8,9^.

Thorough comprehension of the spatial distributions of invasive species is crucial for advancing our understanding of the dynamics and ecological processes of invaded vegetation. This encompasses the dispersal of invasive species, competitive interactions with native flora, and the consequences for ecosystem structure and function^10–12^. Furthermore, it is vital for the development of effective strategies for the protection against invasive species^12,13^. Invasive plants frequently exhibit distinctive spatial arrangements of shoots that reflect their reproductive strategies, resource acquisition patterns, and responses to environmental conditions^11,14^. The examination of these patterns allows for the identification of pivotal elements that contribute to the success of invasive species, including dispersal mechanisms, colonization dynamics, and the formation of dense patches or monocultures. Spatial analysis techniques, such as point pattern analysis and landscape metrics, offer quantitative insights into the distribution and aggregation of invasive species shoots across landscapes^13^. Such analyses provide valuable insights that inform effective management strategies, including targeted eradication efforts, spatial prioritization of restoration activities, and prediction of future invasion trajectories under changing environmental conditions^13^. In addition, the application of RS technologies — encompassing data collected by unmanned aerial vehicles (UAVs) — in conjunction with geographic information systems (GIS) facilitates the real-time mapping and monitoring of invasive species distributions. This, in turn, enables the implementation of early detection and rapid response initiatives^12,15^. Nevertheless, to date, limited research has been conducted on investigating the spatial patterns of plants derived from remote sensing data obtained from drones. This would allow for the revelation of their elementary ecological interactions^16^.

Common milkweed (*Asclepias syriaca* L.) is regarded as one of the most detrimental invasive species by numerous databases, including CABI^17^DAISIE^18^the European Commission^19^GRIIS^20^and EPPO^21^. The original native range of the milkweed is North America, and it was introduced to Europe in 1629^22,23^. Currently, the species is considered one of the most aggressive invasive plants in Hungary. The invasion of common milkweed is most pronounced in plant communities that have already undergone degradation or disturbance due to external factors^24^. The primary conservation issues that arise from this species are related to its potential to impede the regeneration of semi-natural communities in the areas it occupies^24,25^. The plant’s effective vegetative (clonal) growth enables it to spread slowly but persistently even in open sandy grasslands^22,24,26^. The species has caused a substantial transformation of natural vegetation across large areas and continues to threaten the remaining native plant communities. In light of the rising prevalence of degraded areas, milkweed is exhibiting a parallel expansion, occupying these areas^27^. An increasing number of studies are examining the impact of the species’ invasion on natural vegetation, as reviewed by Follak et al.^25^. Nevertheless, there is still much to be learned about its population-level spread dynamics, the formation of shoot density, spatial arrangement (pattern formation), and any potential regularities in these aspects. To better understand its impact on vegetation, it is essential to explore the structure, texture, and related functions and mechanisms. An investigation into the spatial arrangement of milkweed shoots has the potential to significantly enhance our understanding of the species’ spread and dynamics. This, in turn, could facilitate more effective conservation management strategies and enable a more precise assessment of its impact on vegetation^13^. The better understanding and successful control of invasive plant species spreading requires more precise, faster mapping and monitoring methods in large areas^13^.

The objective of this study was to evaluate the effectiveness of UAV-based imagery in detecting and characterising the spatial structure of invasive common milkweed populations. Specifically, we aimed to compare drone-based observations with high-resolution ground surveys in terms of shoot count, spatial distribution metrics, and point pattern classification, to assess the suitability of UAV technology for supporting conservation and management efforts.

## Materials and methods

### Study area

**Fig. 1:**
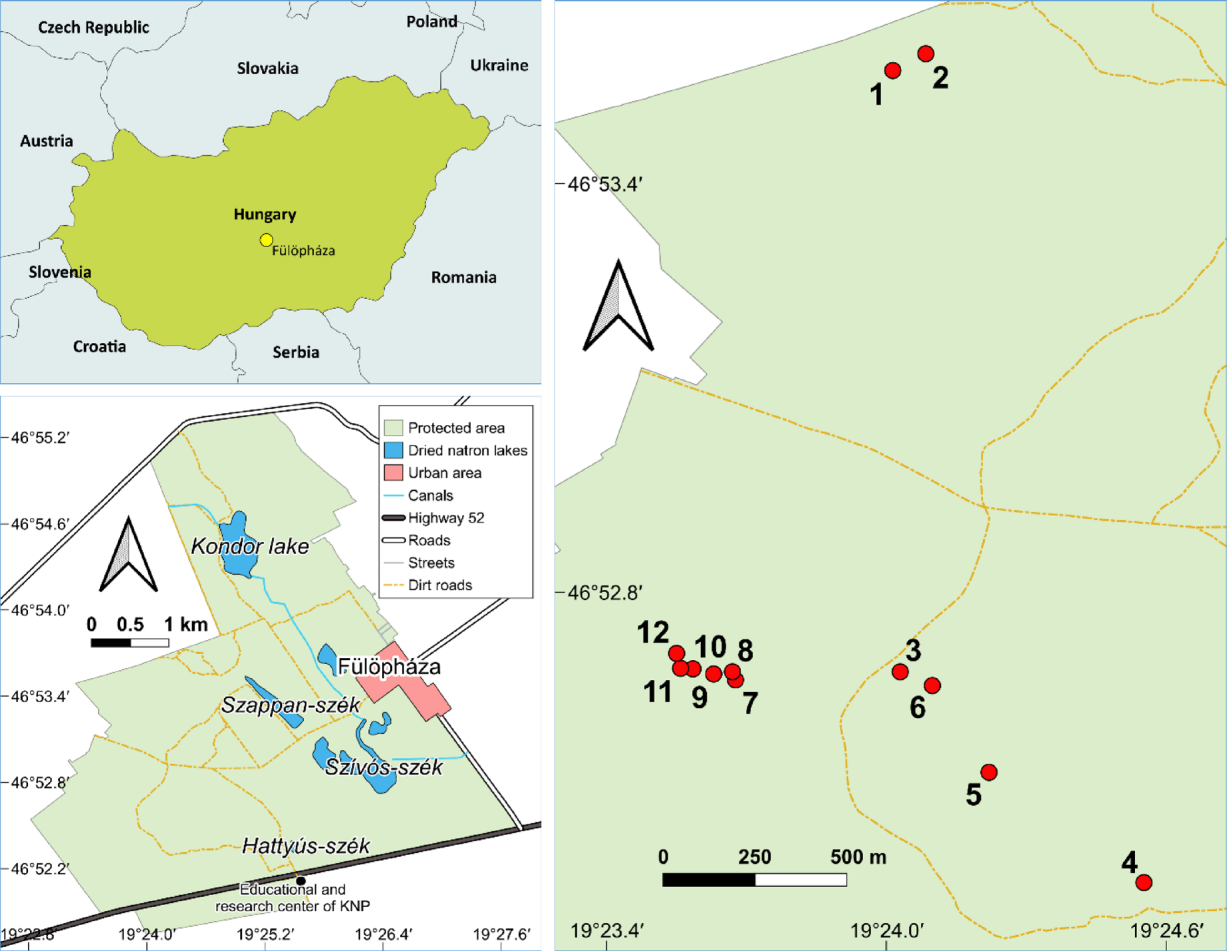
The map depicts the geographical location of the study site and the precise positions of the 12 selected common milkweed stands.

The Fülöpháza Sand Dunes, a protected area situated in the middle of Kiskunság National Park near Fülöpháza, constitutes a part of the UNESCO biosphere reserve (N46°52.92’ E0 19°23.94’; Fig. 1). The area has been under protection since 1974^28^. This area is classified as a Pannonian sand steppe (Natura 2000 code: 6260), which is of particular significance according to the European Union’s Habitats Directive (92/43/CEE)^29^. However, these habitats are facing an existential threat from the proliferation of numerous invasive species^30^ and climate change^31^. The climatic conditions are semi-arid temperate, exhibiting both continental and sub-Mediterranean characteristics^28,29,31^. The mean annual precipitation ranges from 520 to 565 millimeters, while the mean annual temperature fluctuates around 10.33 degrees Celsius. The soil in the area is nutrient-poor and has developed on calcareous sand^28,29,31^.

The protected area exhibits a mosaic vegetation structure, with open sandy grasslands (*Festucetum vaginatae*) representing the dominant vegetation type. However, the area also encompasses closed sandy grasslands, poplar-juniper shrublands, and habitats that have been subjected to human-induced disturbances and modifications^29^. The area is home to a plethora of rare, endangered, and endemic species, including *Festuca vaginata* Waldst. et Kit. ex Willd., *Colchicum arenarium* Waldst. et Kit., *Dianthus diutinus* Kit., *Dianthus serotinus* Waldst. et Kit., and *Iris arenaria* Waldst. et Kit.^28,29^. Furthermore, a number of soil-dwelling lichen species are present, including *Cladonia convoluta* (Lam.) Anders., *C. furcata* (Huds.) Schrad., *C. magyarica* Vain. ex Gyeln., and *C. rangiformis* Hoffm.^28,29,32^.

### Field sampling methods

**Fig. 2:**
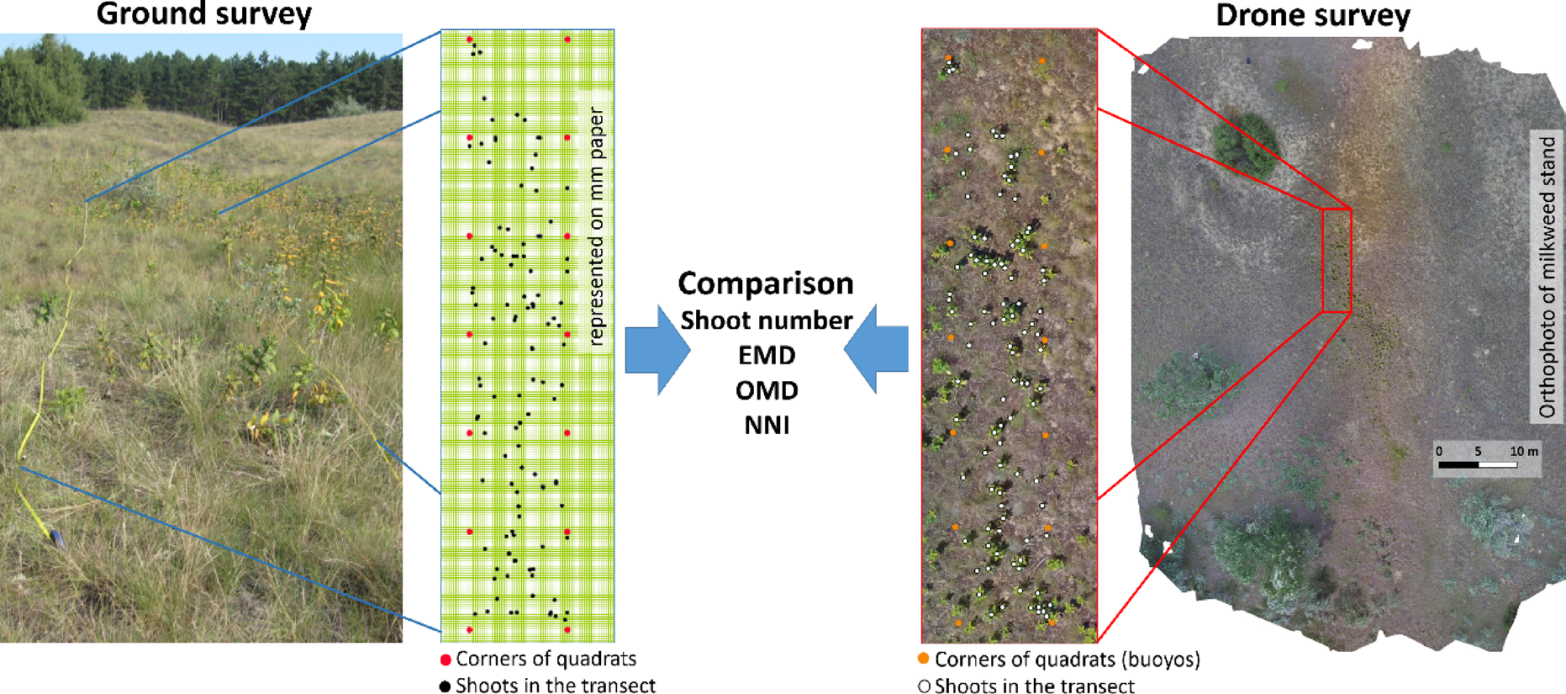
The workflow, conducted on the ground and with the drone, entailed a comparison of the number and spatial pattern of common milkweed shoots. The data were employed for the purpose of spatial pattern analysis. The variables utilized in the analysis were EMD (expected mean distance), OMD (observed mean distance), and NNI (nearest neighbor index).

The spatial point pattern studies were conducted on smaller common milkweed populations. A total of 12 milkweed stands with variable shoot number and density were recorded in the Fülöpháza area of the Kiskunság National Park (Fig. 1). The ground and aerial recordings were conducted on the same day, July 14 and 28, 2020. The preceding days had also been marked by dry and sunny conditions. First, an aerial survey of the target vegetation was conducted, followed by a ground survey (validation) to prevent any anomalies caused by trampling. Consequently, the number of folds and patterns identified in the images captured by the drone can be compared with the findings from the ground-based recording (Fig. 2). The distinguishability of the milkweed is enhanced by the fact that its morphology and even its spectral characteristics diverge from those of the surrounding open sand grassland species^22,33^. Consequently, it can be readily identified with a field resolution of approximately 5 cm through the use of RGB (real color) recording^33,34^.

The aerial photography was conducted using a DJI Phantom 3 Standard drone, manufactured by Da-Jiang Innovations in Shenzhen, China. The camera of the drone is equipped with a three-axis (pitch, roll, yaw) gimbal and features a 1/2.3″ CMOS sensor and an f/2.8 lens. The electronic shutter speed is 8 to 1/8000 of a second, which is optimized for aerial photography. The camera is capable of capturing images with a resolution of 12 megapixels, in both the JPEG and DNG RAW formats. The flight parameters for mapping were established within the DroneDeploy software. In all cases, a ratio of 70–70% was allocated for base direction and transverse overlap. The flight speed was set at 4 m/s, while the flight height was 20 m, which is sufficient to ensure that the target vegetation can be easily recognized while avoiding potential hazards such as bushes and small trees in the area to be surveyed and minimizing interference with air traffic.

To guarantee precision, each stand was additionally documented on the ground in the following manner: In each 12-meter-long transect of a given stand (comprising one row of six contiguous 2-meter-by-2-meter quadrats), the position of each milkweed shoot was recorded with centimeter accuracy, as previously described by Bakacsy and Bagi^13^by manually mapping shoot positions within 2 × 2 m quadrats using measuring tapes for precise spatial coordinates. Marker buoys were positioned in the corners of the 2 m × 2 m quadrats, with three of these utilized as ground control points (GCPs) in each group. To ensure that the three markers used as GCPs did not fall in the same plane, care was taken to select them at random. The GPS coordinates were determined using a Garmin eTrex Legend GPS device (Garmin International Inc., Olathe, KS, USA), which is typically utilized by those engaged in nature conservation management.

The average imaged area per UAV capture was approximately 58.4 m^2^ ± 11.2 m^2^. However, spatial analyses were strictly confined to a 12-meter transect. In certain instances (transects 7–9 and 10–11), multiple transects were located within a single UAV image due to geographic proximity. However, it should be noted that all processing and statistical analyses were conducted independently for each transect. It is noteworthy that all transect regions were covered by a minimum of nine overlapping UAV images, thereby ensuring high spatial accuracy and redundancy in photogrammetric reconstruction.

### Data processing

In order to create orthophotos, the drone photographs were aligned using photogrammetric software Agisoft Metashape Professional (version 1.5.4). The workflow consisted of several automated steps that transform raw overlapping images into georeferenced, high-resolution orthophotos: Photo alignment – Establishes the relative position and orientation of individual images, generating an initial sparse point cloud based on overlapping image features. Dense cloud generation – Produces a high-density 3D point cloud representing surface geometry in detail. Tiled model – Creates a textured three-dimensional surface model using the dense cloud for enhanced visualization of topography. Digital Elevation Model (DEM) – Generates an elevation model derived from the dense point cloud, capturing micro-relief variations. Orthomosaic generation – Produces a geometrically corrected, georeferenced mosaic of the imagery suitable for metric analysis. These steps ensure precise spatial representation of the study transects and enable accurate digitization of individual shoots. The workflow follows widely adopted UAV-based methodologies in ecological and conservation studies^12,15,33,35^. For detailed technical guidance, we refer readers to the Agisoft Metashape documentation and related literature on UAV-based photogrammetry. Once the orthomosaics were generated and georeferenced, they were ready for visual interpretation and digitization of individual shoots.

Subsequently, an independent analyst prepared the orthomosaics for shoot identification. For the UAV-based workflow, the positions of milkweed shoots were manually delineated on orthomosaics using GIS software (ArcMap, version 10.7 for Windows; Redlands, CA). Ground-based survey data were digitized by plotting each shoot’s recorded position from field maps (drawn on millimeter paper for each quadrat) into the GIS environment to ensure precise georeferencing. These datasets were then subjected to spatial point pattern analysis. The expected and observed mean distances (EMD and OMD, in cm) and nearest neighbor index (NNI) were calculated using the Euclidean distance method in ArcMap software (version 10.7 for Windows; Redlands, CA). This was achieved by using the mean nearest neighbor tool (Spatial Statistics) within the Environmental Systems Research Institute (ESRI) suite of software, to obtain a unique value for each transect and method (Fig. 2).

### Workload measurement and system time tracking

In order to assess the practical feasibility of both survey methods, the duration of all work phases was recorded. The measurement of human work time was conducted manually during the course of fieldwork and subsequently during the data processing stage. This was achieved by means of the use of both watch and field notes. The duration of each work phase was estimated using manual time recording. The temporal framework encompassing fieldwork and data processing was meticulously documented through the utilisation of standard mobile phone clocks and field notes, which were systematically recorded by the researchers during both on-site and post-field activities. The system time (for drone image processing) was obtained from the photogrammetry software’s process logs (Agisoft Metashape), which reported the duration of each automated step (e.g., image alignment, dense cloud generation, digital elevation map, and orthomosaic building). The temporal values are expressed in hours and minutes, and presented as mean ± standard deviation (SD), based on the 12 transects.

### Cost structure comparison

The estimated costs for both survey approaches were recorded to facilitate a structured comparison of the required resources. The cost analysis distinguishes between initial equipment costs, recurring per-survey costs and annual system-level costs.

Initial costs for the UAV-based workflow included the acquisition of a drone platform (e.g., DJI Phantom 3 or similar model), photogrammetric processing software (e.g., Agisoft Metashape), and potential computer hardware upgrades. In the case of the ground-based method, only basic field instruments and a GPS device were considered. GIS software was assumed to be freely available (QGIS) or institutionally licensed (ArcGIS). The inclusion of data storage and hardware depreciation was deemed pertinent to the study’s objectives.

The recurring per-survey costs per 12-m transect encompassed the estimated field labour time for two-person teams, calculated at €10–15 per hour per person, in addition to transport, manual data annotation, and maintenance expenses. System processing time for UAV images (approximately 25 h per orthophoto) was considered as fully automated and therefore excluded from labor cost calculations (as it did not necessitate user input).

Finally, annual system-level costs, such as maintenance and depreciation of UAV hardware (e.g., battery replacement, calibration, and insurance), were listed separately from per-survey costs. This category does not apply to ground-based workflows beyond negligible maintenance of basic field gear.

### Statistical analysis

In order to facilitate a comparison of ground-based and drone-captured data, a series of numerical values were employed, including shoot numbers, EMD, OMD, and NNI. The normality of the data was evaluated through the implementation of the Shapiro-Wilk test. The data were compared using Pearson’s correlation when the distribution was normal and Spearman’s rank correlation when the distribution was non-normal. The statistical analysis and visualization of the data were conducted using GraphPad Prism version 8.0.1.244 for Windows (GraphPad Software, La Jolla, California, USA). The smallest statistically significant level is *p* = 0.05. The data were presented in the format of mean ± SD, with a total of 12 elements (stands) surveyed.

In order to assess the spatial point pattern characteristics (i.e., whether these were random, clustered, or dispersed) of milkweed shoots, a nearest neighbour analysis was conducted using ArcMap 10.7. The z-score and associated *p*-value were calculated for each transect to test for deviation from complete spatial randomness (CSR). A z-score near 0 and a high *p*-value (> 0.05) indicated a random pattern, a significantly negative z-score (*p* < 0.05) indicated clustering, and a significantly positive z-score (*p* < 0.05) indicated a dispersed pattern. These values were then utilised to categorise the spatial structure of each transect.

## Results

**Fig. 3:**
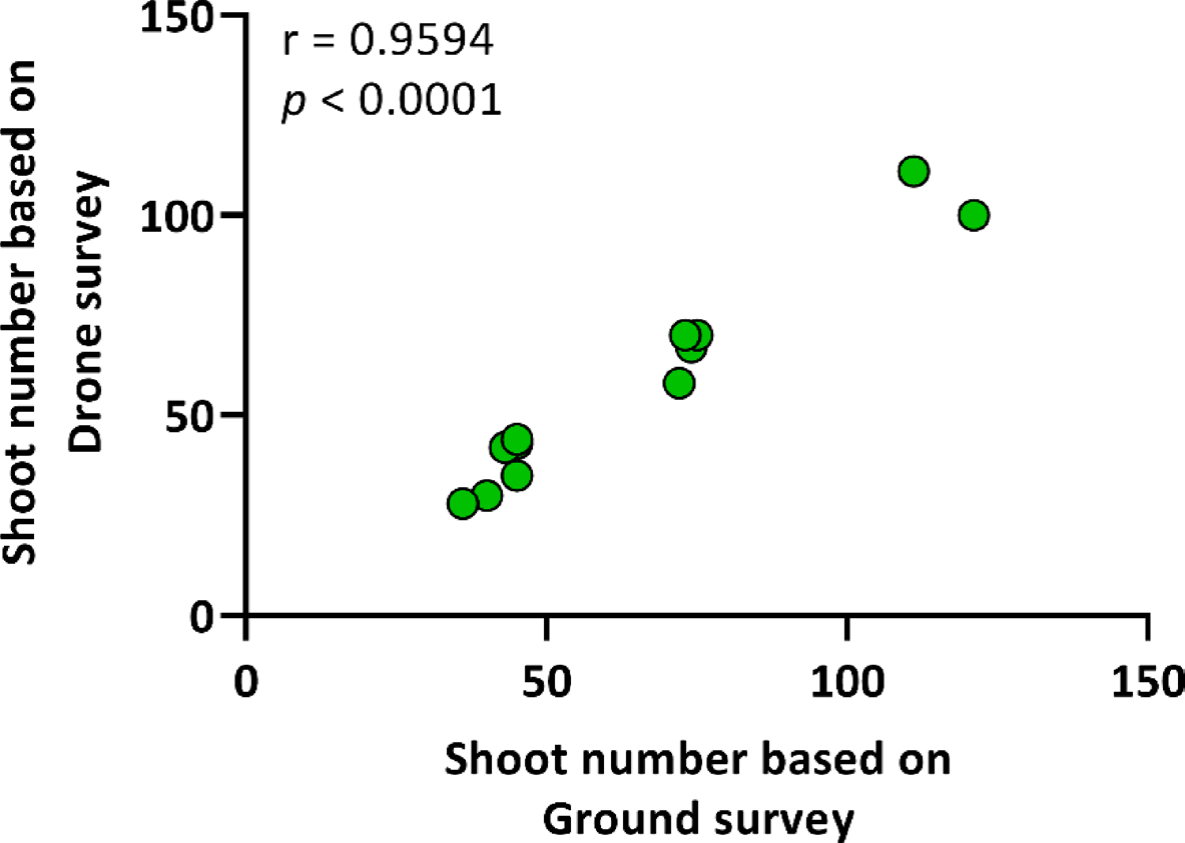
Correlation between the number of common milkweed shoots based on drone and ground survey. A Spearman’s rho correlation (two-tailed) was used, with a sample size of *n* = 12.

A strong positive correlation was observed between the two methods in comparing the number of shoots in the transects (*r* = 0.9594; *p* < 0.0001; Fig. 3). With regard to the ground survey, the mean number of shoots was found to be 65 ± 28.12, while for the drone survey, it was 58.17 ± 26.28 (Table 1).

**Table 1:** The data utilized for the purpose of Spatial pattern analysis are as follows: the expected mean distance (EMD), observed mean distance (OMD), and nearest neighbor index (NNI) were calculated using the Euclidean distance method in arcmap software. A *p*-value of 0.05 was employed as the threshold for statistical significance, with values exceeding this threshold were deemed insignificant, *n* = 12.

Transect No.	Ground survey			
	Number of shoots	EMD (cm)	OMD (cm)	NNI
1	72	27.472	26.842	0.977
2	111	22.806	19.745	0.866
3	121	21.145	19.613	0.928
4	74	28.380	30.851	1.087
5	45	36.056	32.356	0.897
6	53	32.014	30.820	0.963
7	46	33.935	33.170	0.977
8	38	33.850	28.210	0.833
9	43	35.509	38.763	1.092
10	45	31.686	33.012	1.042
11	75	23.423	20.229	0.864
12	73	27.610	28.921	1.047
Mean ± SD	65 ± 28.12	29.85 ± 5.428	28.87 ± 6.206	0.9638 ± 0.088
1	58	31.929	35.509	1.112
2	111	22.913	22.019	0.961
3	100	24.382	25.382	1.041
4	67	30.602	33.240	1.086
5	43	36.533	35.981	0.985
6	35	39.693	43.718	1.101
7	30	40.732	40.486	0.994
8	28	37.850	37.103	0.980
9	42	39.811	47.542	1.194
10	44	32.555	35.718	1.097
11	70	25.462	23.562	0.925
12	70	26.992	31.212	1.156
Mean ± SD	58.17 ± 26.68	32.45 ± 6.463	34.29 ± 7.821	1.052 ± 0.084

**Fig. 4:**
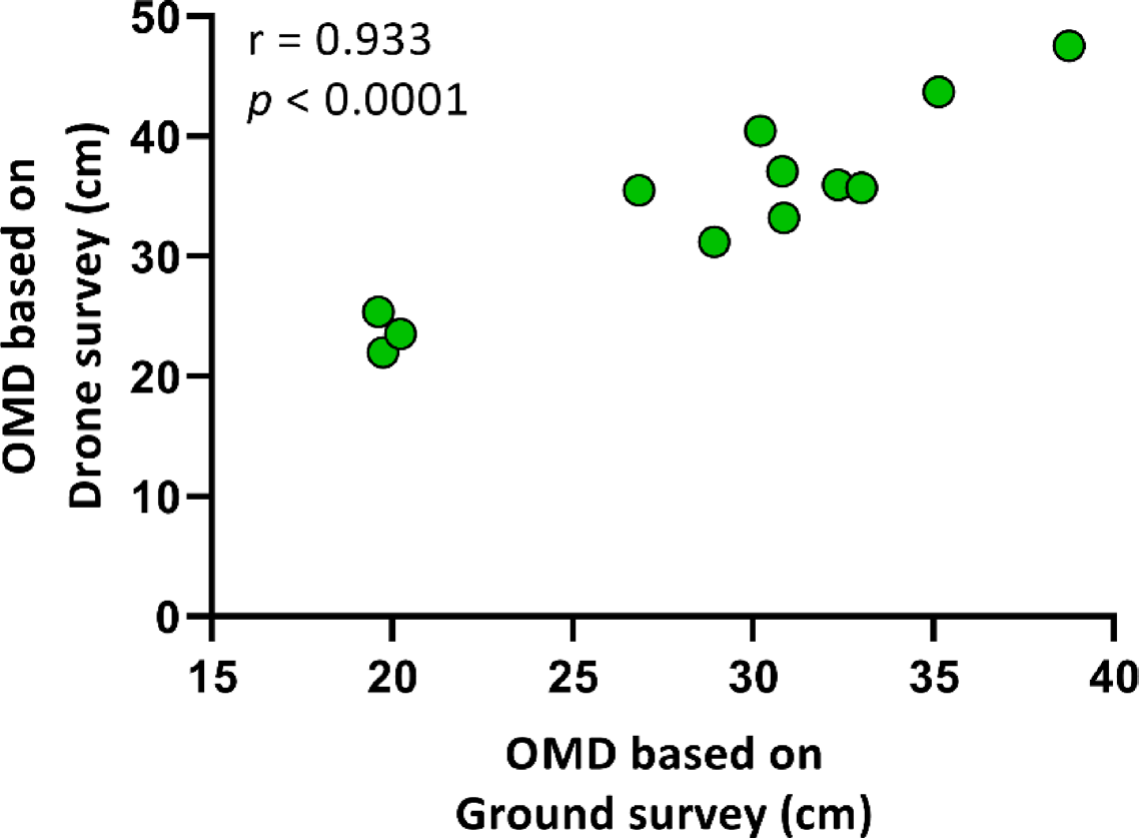
Correlation between the expected mean distance (EMD) of common milkweed shoots based on drone and ground surveys. A Pearson’s r correlation (two-tailed) was used, with a sample size of *n* = 12.

In the case of expected mean distance, the mean distance of the shoots observed during ground-based surveying was 29.85 ± 5.428 cm, while for drone surveying it was 32.45 ± 6.463 cm (Table 1). Nevertheless, a robust positive correlation was also discerned between the two methodologies, with a correlation coefficient of *r* = 0.951, and *p* < 0.0001 (Fig. 4).

**Fig. 5:**
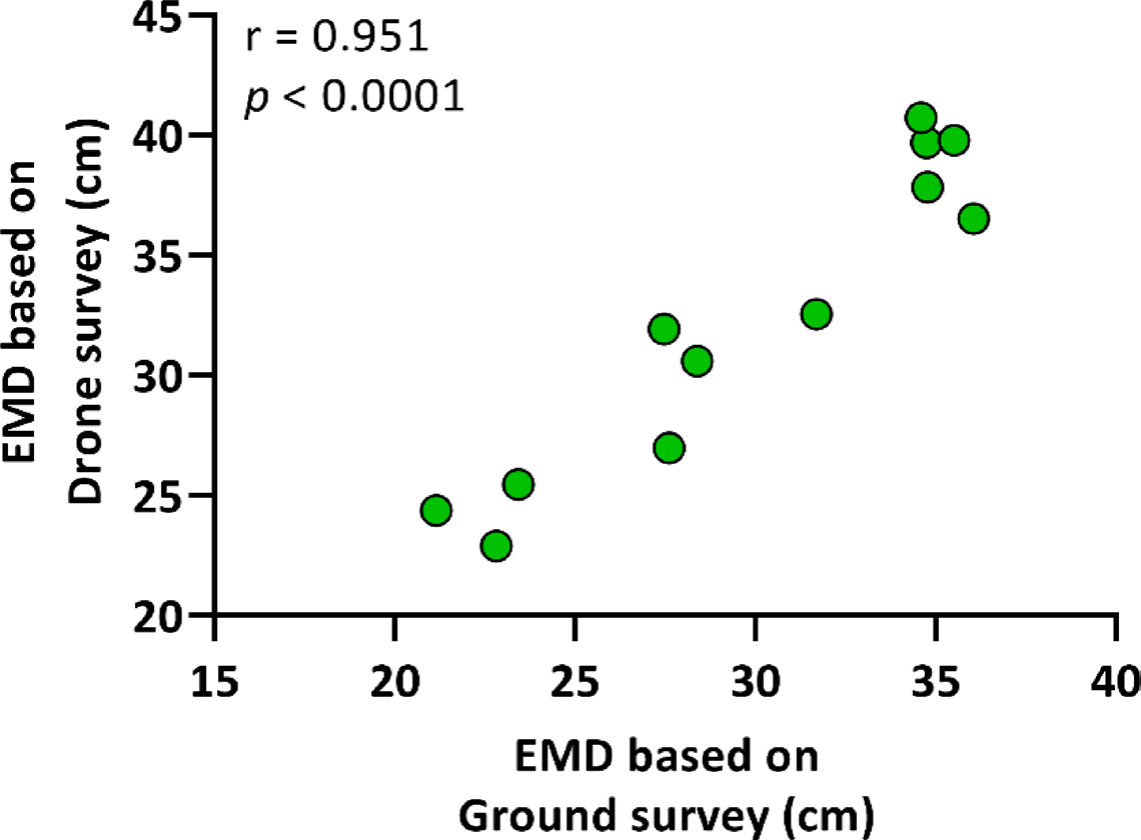
Correlation between the observed mean distance (OMD) of common milkweed shoots based on drone and ground surveys. A Pearson’s r correlation (two-tailed) was used, with a sample size of *n* = 12.

The observed mean distance between the shoots was 28.87 ± 6.206 cm during ground-based surveying and 34.29 ± 7.821 cm with drone surveying (Table 1). As with the EMD, the two surveying methods demonstrated a robust positive correlation (*r* = 0.933, *p* < 0.0001; Fig. 5).

**Fig. 6:**
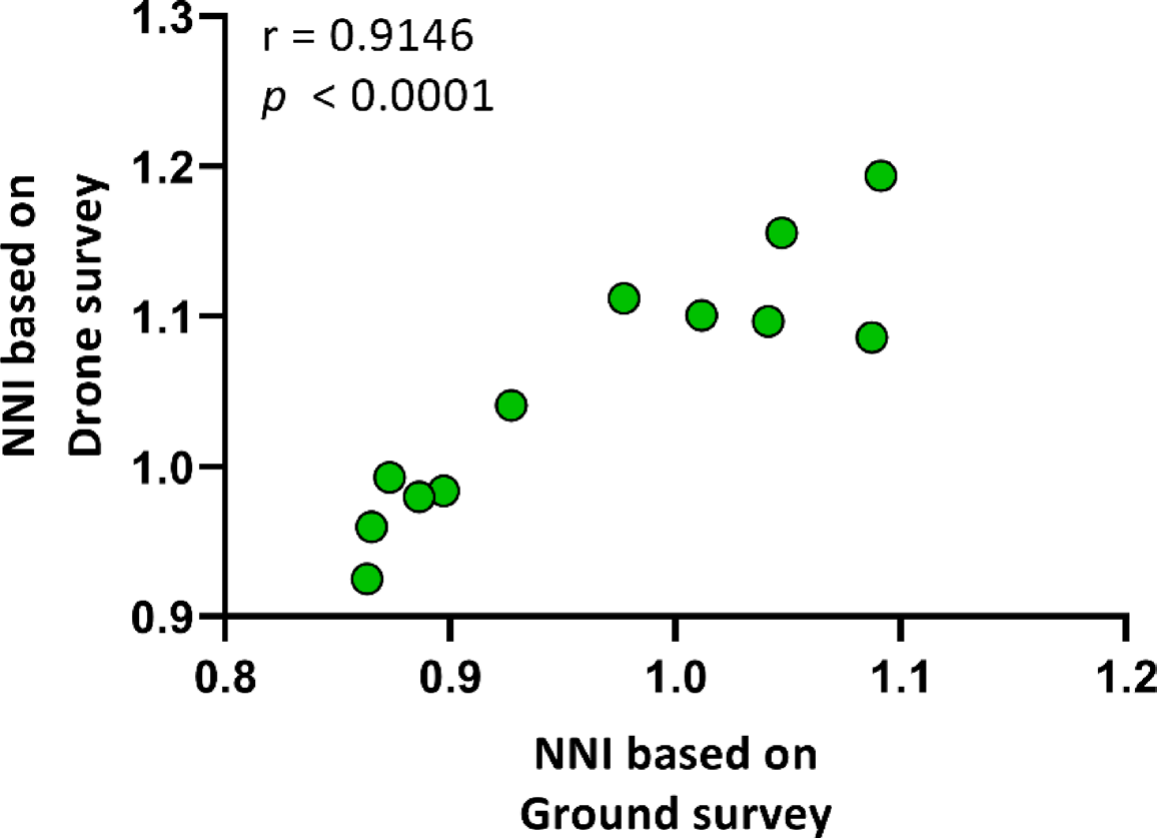
Correlation between the nearest neighbor index (NNI) of common milkweed shoots based on drone and ground surveys. A Pearson’s r correlation (two-tailed) was employed, with a sample size of *n* = 12.

The nearest neighbor index of the shoots obtained through ground-based surveying was 0.963 ± 0.088, while for drone surveying it was 1.052 ± 0.084 (Table 1). Furthermore, a significant positive correlation was identified between the nearest neighbor index of the shoots obtained from the two surveying methods, with a correlation coefficient of *r* = 0.9146, and *p* < 0.0001 (Fig. 6).

The data obtained from the ground-based observations revealed that the arrangement of the shoots exhibited a random pattern on nine occasions. The analysis of drone images identified a random point pattern in seven instances among the transect cases. Two instances were observed to exhibit a random dispersed exchange (Table 2). The results of the ground-based observations indicated the presence of clusters in three transects, which were subsequently identified as random occurrences based on the analysis of the drone images (Table 2).

**Table 2:** Classification of Spatial point patterns of common milkweed shoots based on nearest neighbor analysis (NNI) using data from drone and ground surveys across 12 transects. The analysis was conducted in arcmap (version 10.7) using Euclidean distance to calculate nearest neighbor z-scores and corresponding* p*-values. A significance threshold of *p* = 0.05 was used; values above this were considered statistically non-significant (i.e. random pattern).

Transect No.	Ground survey			Drone survey		
	z-score	*p*-value	Point patterns	z-score	*p*-value	Point patterns
1	−0.373	0.710	random	1.633	0.102	random
2	−2.705	0.007	clustered	−0.787	0.431	random
3	−1.524	0.127	random	0.785	0.433	random
4	1.433	0.152	random	1.350	0.177	random
5	−1.317	0.188	random	−0.190	0.850	random
6	−0.519	0.603	random	1.148	0.251	random
7	−0.292	0.770	random	−0.063	0.950	random
8	−1.965	0.049	clustered	−0.200	0.842	random
9	1.150	0.250	random	2.408	0.016	dispersed
10	0.537	0.591	random	1.233	0.217	random
11	−2.259	0.024	clustered	−1.194	0.232	random
12	0.776	0.438	random	2.502	0.012	dispersed

The total human working time required to complete the ground-based survey was approximately 3 h and 30 min ± 55 min per transect, while the drone-based workflow required approximately 1 h and 30 min ± 10 min (Table 3). The duration of field survey activities was 1 h 58 min ± 47 min for the ground method and 8 min ± 4 min for the drone method. Furthermore, UAV image acquisition was followed by automated photogrammetric processing, including photo alignment (1 h and 17 min ± 55 min), dense cloud generation (23 h 4 min ± 17 h and 27 min), tiled model construction (20 min ± 15 min), digital elevation model (DEM) generation (1 min ± 1 min), and orthomosaic generation (8 min ± 5 min) (Table 3). The total duration of these processing steps was 24 h and 50 min, with a standard deviation of ± 18 h and 43 min. Notably, 23 h of this duration comprised fully automated operations. The manual identification of shoot positions took 25 min ± 8 min for the ground method and 14 min ± 7 min for the drone-based method. Spatial pattern analysis was completed within an approximate time frame of 10 min per transect in both cases (Table 3).

**Table 3:** Estimated duration of work phases in the ground-based and drone-based survey methods, expressed as mean ± standard deviation (SD), *n* = 12. System time refers to automated steps and does not involve human labor.

Work phase		Ground survey	Drone survey
Field operations	Field preparations	~ 1 h	~ 1 h
	Field survey	1 h 58 min ± 47 min	8 min ± 4 min
Drone image processing	Align photos (automated)	–	1 h 17 min ± 55 min
	Build dense cloud (automated)	–	23 h 4 min ± 17 h 27 min
	Build Tiled Model (automated)	–	20 min ± 15 min
	Build DEM (automated)	–	1 min ± 1 min
	Build Orthomosaic (automated)	–	8 min ± 5 min
Data processing	Spatial digitization of shoots	25 min ±8 min	14 min ± 7 min
	Shoot spatial analysis	~ 10 min	~ 10 min
Total human work time		~ 3 h 30 min ± 55 min	~ 1 h 30 min ± 10 min
Total system time (inc. processing)		~ 1 h	~~24 h 50 min ± 18 h 43 min (23 h automated)

The comparative analysis of costs revealed substantial differences between the two survey approaches (Table 4). The UAV-based workflow incurred higher initial investments (€2200–3700) compared to the ground-based method (€600–1600), primarily due to the acquisition of a drone platform and photogrammetric software. However, recurring per-survey costs were substantially lower for UAV surveys, averaging approximately €33–44 per transect, compared to €80–100 for ground-based surveys. This reduction was mainly attributable to shorter field labor and faster spatial digitization of shoots (€3–4 vs. €5–7; Table 4). Although UAV image processing required significant system time (~ 25 h), this phase was fully automated and did not add to labor expenses. Annual system-level costs for UAV maintenance and depreciation were estimated at €200–300, which become negligible when amortized over multiple surveys. Overall, these findings indicate that while UAV monitoring requires greater upfront expenditure, it achieves long-term cost efficiency, particularly in large-scale or repeated applications (Table 4).

**Table 4:** Estimated cost components associated with ground-based and UAV-based milkweed surveys (expressed in euros). Initial costs include equipment and software required for system setup. Recurring costs are calculated per 12-m transect per survey. Maintenance and depreciation represent annual system-level costs, separated from per-survey estimates. Costs are approximate and based on market prices in 2024 in central europe.

Cost category /component	Ground-based survey	UAV-based survey	Notes
Initial equipment costs	€600–1600	€2200–3700	UAV: drone (€1200–1800), software (€800–1500), PC upgrade (if needed)
GPS device	€200–400	€200–400	Required for GCP/ground truthing
Basic field gear	€300–600	€300–600	Identical (tape, markers, log sheets)
UAV acquisition	–	€1200–1800	DJI Phantom 3 or similar entry-level drone
Photogrammetry software	–	€600–1500	Agisoft Metashape, Pix4D, etc.
Data storage/PC upgrade	–	€300–800	Depending on processing capacity
GIS software	€0 or €1500–2000/year	€0 or €1500–2000/year	QGIS or ArcGIS
Recurring per-survey costs (est. per 12-m transect)	**Ground-based survey**	**UAV-based survey**	**Notes**
Field time (2 staff)	~ 3.5 h (€70–90)	~ 1.5 h (€30–40)	Based on €10–15/hour/person
Transport & field logistics	~€10	~€10	Similar for both methods
Spatial digitization of shoots	~ 25 min (€5–7)	~ 15 min (€3–4)	Faster in UAV due to visual clarity
System time (automated)	Negligible	~ 25 h (0 labor)	No cost unless computing billed
Annual system-level costs	**Ground-based survey**	**UAV-based survey**	**Notes**
Maintenance & depreciation	Minimal	€200–300/year	UAV battery, calibration, insurance

## Discussion

The present study demonstrated that UAV-based monitoring can serve as a reliable and efficient method for detecting and quantifying the spatial structure of invasive plant populations at the shoot level. The findings demonstrated a robust correlation between drone-based and field-based data with respect to shoot number, spatial metrics, and distribution patterns, thereby substantiating the efficacy of low-cost RGB imagery in the context of ecological research and prospective conservation applications.

The number of milkweed shoots detected via drone was slightly lower than ground-based counts, yet a strong positive correlation was found between the two (Fig. 3). The underestimation may be attributed to the fact that milkweed shoots can occasionally overlap, given that the morphology and spectral characteristics of the target species are markedly distinct from those of the surrounding vegetation. This phenomenon has been corroborated by other studies, which have similarly indicated that aerial surveys may occasionally underestimate the number of plants. Bakacsy et al.^33^ successfully distinguished shoots using RGB indices, though obscuration remained an issue. Papp et al.^36^ corroborated these challenges by employing a multispectral convolutional neural network. This can be a significant challenge, particularly in the context of dense populations of invasive species. The inherent limitations of drone-based data collection, such as the potential for error, may lead to an increase in the margin of error in the derived data, making it more challenging to accurately determine the precise spatial distribution of the target species. These findings are also reflected in the results of the present study, which indicate that while the strongest positive correlation was observed in the number of shoots, this correlation weakened somewhat (but remained significant and positive) for the studied spatial pattern parameters.

With regard to the observed misclassification of spatial point patterns in five out of twelve transects, it is important to note that the discrepancies largely arose in cases where the z-scores of ground-based surveys were close to the threshold for statistical significance. This finding indicates that minor variations in the detection of shooting events (e.g., missed or merged individuals due to partial occlusion in aerial images) have the potential to alter a transect’s classification from clustered to random or vice versa. While this underscores a shortcoming of drone-based pattern analysis at the fine scale, it does not in any way compromise the method’s overall reliability, particularly when the objective is to identify broader-scale patterns or to prioritise high-density patches for management. Furthermore, pattern classification is inherently sensitive to subtle changes in spatial input, and the combination of UAV-based surveys with ground truthing remains the optimal approach when detailed spatial information is imperative for effective decision-making.

Despite this, the present study identified a robust correlation between ground and drone data for spatial pattern parameters such as EMD, OMD (Figs. 4 and 5), and NNI (Fig. 6), indicating that drone imagery is effective in assessing milkweed distribution. In a similar vein, Lam et al.^37^ attained a 92.1% detection accuracy rate by employing a deep learning model to map *Rumex obtusifolius* L., yet due to misclassifications, the F1 score was 78.7%, underscoring the necessity for model refinement and the generation of high-quality training data. The observations made by the aforementioned researchers revealed that dense stands were underestimated due to the presence of overlapping vegetation. It is recommended that future research endeavours consider integrating deep learning with spatial analysis as a means to enhance the reliability of detection, particularly in the context of rapidly propagating species that exert significant ecological influence^12^. The results obtained from this study provide a robust foundation for the development of drone-based monitoring methodologies for the study of milkweed’s annual dynamics and large-scale spatial mapping, with particular relevance to areas that are difficult to access.

From a practical standpoint, UAV-based monitoring represents a valuable addition to the invasive species management toolkit. Although the technology involves a more complex setup and post-processing workflow, it dramatically reduces the amount of field labor—often the most time-consuming and logistically challenging part of vegetation surveys. It is noteworthy that the time taken for manual delineation and analysis was comparable to that of field-based work, however, drone-assisted data collection was considerably faster (Table 3). This makes drone monitoring particularly suitable for large areas, repeated surveys, or difficult-to-access habitats. Comparable conclusions have been drawn in previous studies, including those by Müllerová et al.^12^Bolch et al.^15^and Dao et al.^38^which emphasized the repeatability, scalability, and labor-saving potential of UAV-based workflows in invasive plant monitoring. This conclusion is further supported by Barbizan Sühs et al.^39^who demonstrated that UAV surveys for detecting and controlling invasive *Pinus* spp. in a subtropical coastal ecosystem were not only significantly more time-efficient but also markedly more cost-effective. In a manner analogous to the present study, their research underscores the significance of landscape openness and species morphology in facilitating precise visual detection through the utilisation of RGB imagery alone, obviating the necessity for more sophisticated multispectral or hyperspectral sensors. A cost comparison between the two survey methods revealed clear differences in the present study. The UAV-based workflow necessitated a higher initial investment, ranging from €600 to €1,600 (Table 4). However, fieldwork time was significantly reduced with the use of UAVs, with an average of approximately 1.5 h per transect recorded as opposed to 3.5 h. Furthermore, the majority of the approximately 25-hour image processing was automated and did not require human input. These findings are consistent with those of previous studies, which found that UAV-based approaches, though initially more costly, offer greater efficiency in long-term or large-scale settings^12,16,37^. The GIS processing in our study was performed in ArcMap, but it is possible to replicate similar workflows using QGIS, a free and widely adopted alternative^33^. Given that GIS operations take place after the survey, the choice of software has minimal impact on pre-transect time, although it may have a significant effect on overall project cost. The cost estimates derived from this study are in alignment with those previously reported by Barbizan Sühs et al.^39^who found that the utilisation of drones for the detection and control of invasive pine trees resulted in a reduction of total costs by approximately one-third in comparison with conventional methods, and a significant decrease of over 85% in field time. Despite the differences in species and habitat, a comparable pattern emerged: a higher equipment cost was offset by a reduction in labour.

Local environmental conditions and interspecies competition play key roles in shaping spatial patterns. Dao et al.^38^ demonstrated that high-resolution hyperspectral UAV imagery can effectively map the distributions of native and invasive plants in Canadian grasslands. Their findings demonstrated that invasive species such as *Bromus inermis* Leyss. and *Dactylis glomerata* L. dominate drier, hilly regions characterised by a paucity of native competitors, while native species such as *Solidago canadensis* L. and *A. syriaca* are more prevalent in wetter, flat areas. Similarly, Erfanifard et al.^16^ emphasized the potential of drone-based remote sensing to analyze spatial distribution and species interactions (e.g., competition, exclusion, facilitation), though such studies remain limited. Nevertheless, this information would also be particularly useful in the case of interactions between invasive species and natives. Consequently, these techniques could facilitate an understanding of the mechanisms by which invasive species gain a competitive advantage over certain types of vegetation and which native species they threaten.

In this study, the observed shoot densities ranged from approximately 1.6 to 5 individuals per square meter, which is characteristic of medium-density stands in open sand grasslands of the Kiskunság region. It is acknowledged that, in accordance with field experience and previous investigations, both lower and substantially higher densities can occur^13,24,25^. In areas with very sparse populations, the efficacy of UAV detection is anticipated to be amplified, a phenomenon attributable to diminished overlap and augmented shoot visibility. However, in highly dense vegetation, the process of detecting shoots from vertical imagery can be hindered by the presence of overlapping foliage and occlusion effects, resulting in underestimation. Indeed, the present study already suggests that a certain degree of undercounting may occur under such conditions. Thus, while the present UAV-assisted workflow has been shown to be resilient in typical density ranges, its application under more extreme conditions may benefit from further optimization. Potential areas for optimisation include the use of oblique imagery, increasing flight resolution, or employing automated detection algorithms^33,34,36^.

It is important to note that the present study was conducted in open sandy grasslands, where *A. syriaca* exhibits a distinctive morphology and coloration that contrasts sharply with the background vegetation. The utilisation of RGB imagery^33^ proved instrumental in the identification of individual shoots. In habitats characterised by greater structural complexity or species richness (e.g., forest edges, shrublands, or tall-grass meadows), the detectability of milkweed shoots may be reduced due to increased spectral and structural overlap with other species. In such cases, the use of additional sensors (e.g., multispectral or hyperspectral cameras), variable viewing angles, or machine learning-based object detection may be necessary to maintain accuracy^27,33,36^. Consequently, while the present findings validate UAV-based monitoring in visually distinct habitats, further adaptations would be required for broader habitat applicability.

Despite the modest spatial extent of the sampling units employed (12 m transects), the primary objective of this study was not to directly inform operational-scale management mapping. Instead, it was to evaluate the feasibility and accuracy of UAV-based methods in capturing the fine-scale spatial structure of invasive populations. It is acknowledged that management decisions in the field of conservation biology frequently necessitate assessments that encompass extensive geographical areas. However, the development of effective strategies for broad-scale application is contingent upon a robust understanding of the underlying spatial dynamics of invasive species at the local scale. The analysis of fine-resolution point pattern data, such as that presented here, can offer valuable insights into the behaviour of clonal spread, aggregation, and vegetative regeneration processes. These processes are often masked at broader scales. Moreover, the findings of this study provide a robust proof-of-concept for the reliability of drone imagery in capturing shoot-level distribution metrics under field conditions. This methodological validation is imperative before the deployment of UAV-based systems for large-area mapping. By confirming the strong correlation between spatial metrics derived from drone data and those obtained from ground surveys, this study provides a foundation for the upscaling of drone-based monitoring of *A. syriaca* patches in open landscapes. Such scaling could then support practical conservation applications, including post-treatment monitoring and prioritization of control efforts in extensive habitats^12^. For instance, Bakacsy and Bagi^13^ examined the regeneration of herbicide-treated milkweed over a period of seven years using fine-scale spatial analysis, a method analogous to ground methods applied in this study. The findings of the study indicated that the treatment in question resulted in the disruption of the species’ stable spatial pattern and a consequent reduction in pod production. However, by the second year post-treatment, localized thickenings reappeared and became new centers of spread^13^. This finding is consistent with the results obtained in this study, which demonstrate that drone-based spatial pattern analysis is an effective method for identifying areas of regeneration. The insights gained from this fine-scale UAV study provide a valuable methodological basis and ecological context for future applications in broader conservation strategies.

## Conclusion

The study effectively demonstrates that unmanned aerial vehicle (UAV) imaging can be a valuable tool in examining the spatial patterns of invasive common milkweed populations. The strong positive correlations between the UAV-derived data and ground survey data for various spatial metrics validate the reliability of drone imagery in detecting and analyzing the spatial organization and population size of invasive species. However, the study does highlight some limitations, such as the occasional underestimation of shoot numbers in drone imagery due to overlapping vegetation and potential obscuration. This suggests that while UAVs are effective, they may not fully capture dense or complex vegetation structures, potentially leading to an incomplete assessment of invasive species spread.

Future research should focus on refining UAV imaging techniques to address these limitations, possibly by integrating advanced sensors or machine learning algorithms that can better differentiate between overlapping or closely situated shoots. Additionally, further studies could explore the application of UAVs in different environmental contexts or for other invasive species to determine the generalizability of the findings.

Overall, UAVs show promise as a tool for monitoring invasive species, aiding in conservation efforts by providing detailed spatial data that can inform targeted management strategies. However, their effectiveness may vary depending on the complexity of the vegetation and the density of the invasive species being studied.

## Data Availability

The data that support the findings of this study are available from the corresponding author upon reasonable request.
